# Off the Clock: From Circadian Disruption to Metabolic Disease

**DOI:** 10.3390/ijms20071597

**Published:** 2019-03-30

**Authors:** Eleonore Maury

**Affiliations:** Endocrinology, Diabetes and Nutrition Unit, Institute of Experimental and Clinical Research, Faculty of Medicine, Université Catholique de Louvain (UCLouvain), B-1200 Brussels, Belgium; eleonore.maury@uclouvain.be; Tel.: +32-2764-9576

**Keywords:** obesity, molecular clock, circadian rhythm, metabolism, adipose tissue, suprachiasmatic nucleus, high-fat diet, nutrients

## Abstract

Circadian timekeeping allows appropriate temporal regulation of an organism’s internal metabolism to anticipate and respond to recurrent daily changes in the environment. Evidence from animal genetic models and from humans under circadian misalignment (such as shift work or jet lag) shows that disruption of circadian rhythms contributes to the development of obesity and metabolic disease. Inappropriate timing of food intake and high-fat feeding also lead to disruptions of the temporal coordination of metabolism and physiology and subsequently promote its pathogenesis. This review illustrates the impact of genetically or environmentally induced molecular clock disruption (at the level of the brain and peripheral tissues) and the interplay between the circadian system and metabolic processes. Here, we discuss some mechanisms responsible for diet-induced circadian desynchrony and consider the impact of nutritional cues in inter-organ communication, with a particular focus on the communication between peripheral organs and brain. Finally, we discuss the relay of environmental information by signal-dependent transcription factors to adjust the timing of gene oscillations. Collectively, a better knowledge of the mechanisms by which the circadian clock function can be compromised will lead to novel preventive and therapeutic strategies for obesity and other metabolic disorders arising from circadian desynchrony.

## 1. The Mammalian Molecular Clockwork: A Short Story

**Figure 1:**
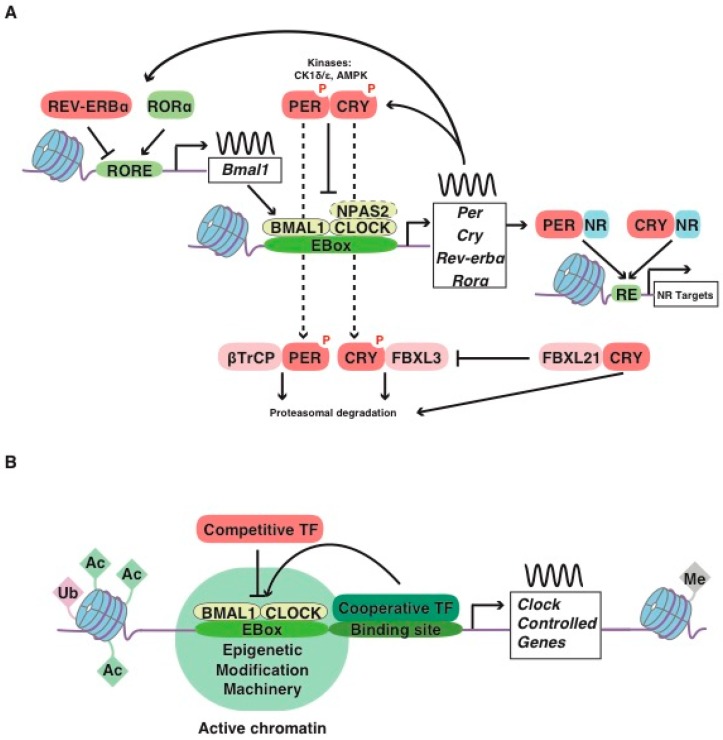
(**A**) Core molecular clock network. The mammalian circadian clock consists of transcription/ translation feedback loops in which the positive limb (CLOCK or NPAS2 and BMAL1) heterodimerizes and activates the transcription of downstream genes, including *Per*, *Cry*, *Ror*α and *Rev-erb*α. The negative limb proteins (PERs and CRYs) multimerize and inhibit CLOCK/BMAL1. The phosphorylation of the repressors mediated by Casein Kinases 1 (*CK1ε* and *CK1δ*) and AMPK directs these proteins to ubiquitin-mediated proteasomal degradation (which, for CRY1, is regulated by FBXL protein ratios). In a secondary loop, *Bmal1* is regulated by the repressor REV-ERBα and its opposing nuclear receptor RORα, which bind competitively to the shared element RORE, thus repressing or activating the transcription of the *Bmal1* gene, respectively (reviewed in [5]). An additional level of circadian regulation exists with CRYs and PERs, which have been reported to bind independently of other core-clock factors to genomic sites enriched with Nuclear Receptor (NR) recognition motifs (response element, RE) [6,7,8,9]. Post-translational modifications of core-clock factors can also regulate transcription (e.g., deacetylation of BMAL1 or PER2 by SIRT1 [10,11]). (**B**) Regulation of clock-controlled genes. Cell-type and tissue-specific transcription factors bind to specific enhancers and/or respond to specific environmental signals and to chromatin accessibility (regulated by covalent modifications of DNA and histone tails, including histone acetyltransferases such as CBP [12,13], histone deacetylases such as SIRT6 [14], the histone lysine demethylase JARID1a [15], and the histone methyltransferase MLL3 [16], which activate transcription). Transcription factors can compete for CLOCK/BMAL1 binding (e.g., bHLH-ZIP factor USF1 [17], inflammatory factor NF-κB [18], nuclear receptor HNF4A [19], MYC oncogenic factor [20] or through chromatin remodeling (for example the recruitment of the HDAC3 co-repressor by REV-ERBα to regulate BMAL1 binding [21]) or through cooperative effects (lineage-determining transcription factors such as PDX1 [22] or hypoxia-induced HIF1α act synergistically with BMAL1 [23]), thus potentiating or inhibiting the metabolic outputs.

Circadian timekeeping is a ubiquitous feature of most organisms that allows appropriate temporal regulation of an organism’s internal metabolism to anticipate and respond to recurrent daily changes in the environment [1]. In most organisms, conserved transcriptional feedback loops (molecular clocks) are the core mechanism of circadian timekeeping. Biological clocks control behavior and physiology through a transcription-translation feedback loop encoded by activators [helix-loop-helix PER-ARNT-SIM transcription factor (bHLH-PAS); CLOCK/BMAL1 or the brain paralog NPAS2/BMAL1; BMAL1 also known as ARNTL] and repressors (PER/CRY) present in brain and peripheral cells. In mammals, CLOCK/BMAL1 heterodimers drive the daytime expression of three *Period* (*Per1-3*) and two *Cryptochrome* (*Cry1-2*) genes through E-box regulatory sequences [2,3] (Figure 1A). After transport to the nucleus, PER/CRY protein complexes, in turn, repress CLOCK/BMAL1 activity during circadian night, thus creating a negative feedback loop. PER/CRY is progressively degraded, allowing for a new cycle (Figure 1A). CRY1 itself is able to bind to the transactivation domain (TAD) of BMAL1 at sites that overlap with the binding sites of the coactivator, histone acetyltransferase CREB-binding protein (CBP), and its homolog p300, to switch off transcriptional activation by CLOCK/BMAL1 [4]. Notably, BMAL1 also has a conserved paralog, BMAL2, that is unable to rescue the circadian rhythm in *Bmal1*-knockout (KO) fibroblasts [4].

The reverse of c-erbA α and β (REV-ERB), encoded by *Nr1d1* and *Nr1d2*, and the retinoic acid-related orphan receptor α and β (ROR), encoded by *Rora* and *Rorb* genes, are members of a subfamily of nuclear hormone receptors regulated by heme and oxysterols, respectively. The REV-ERB/ROR proteins are well-known for their role in metabolism and also participate in the core loop, highlighting the intimate connection between circadian rhythm and metabolic transcription [24,25]. The coordinated action of the transcriptional repressor REV-ERBα and the activator RORα creates a modulatory transcriptional feedback loop that involves the rhythmic transcriptional regulation of clock-core genes, such as *Bmal1* [21] (Figure 1A). REV-ERBα also regulates metabolic genes primarily by recruiting the histone deacetylase 3 (HDAC3) corepressor to sites to which it is tethered by cell-type-specific transcription factors [21]. In particular, REV-ERBα binding sites are common in the BMAL1 cistrome [24], which might be explained by an interaction between these proteins [26]. CLOCK/BMAL1 heterodimers drive the expression of thousands of clock-regulated genes that control a vast array of biological processes such as metabolism [27], although the binding of BMAL1 to specific enhancers depends on cell-type-specific transcription factors and regulators [22] (Figure 1B). Moreover, CRYs and PERs can exert supplementary regulatory actions independent of other core-clock factors, namely through the involvement of Nuclear Receptors (NR), including the glucocorticoid receptor, adding a layer of complexity to this simplified model (e.g., [6,7,8,9,28]) (Figure 1A). 

To regulate the transcription of clock-controlled genes, it has been reported that CLOCK/BMAL1 acts as a pioneer transcription factor and can bind nucleosomes to promote rhythmic chromatin accessibility, a process that allows the binding of other transcription factors adjacent to CLOCK/BMAL1 ([29,30], and reviewed in [5]). CLOCK and BMAL1 promote chromatin modifications by recruiting histone-modifying enzymes, histone acetyltransferases p300 and CBP, to core-clock genes, and the resulting euchromatin can facilitate transcriptional activation [13,31] (Figure 1B). Additional modifications involve histone deacetylases, such as the sirtuins SIRT1 [10,11,32,33] and SIRT6 [14], histone lysine demethylases including JARID1a [15], and histone methyltransferases (MLLs) [16] (Figure 1B). In addition to direct modifications of DNA or histone tails, the physical proximity between different regulatory sequences further contributes to the regulation of 24 h gene expression. Recent chromosome conformation capture experiments highlighted the role of epigenetic modifications in chromatin machinery and chromosomal organization in the regulation of clock-controlled gene expression [34], revealing that REV-ERBα is a key molecular player [35]. Indeed, REV-ERBα opposes the enhancer-promoter loop formation through the recruitment of nuclear receptor corepressor and HDAC3 to actively repress rhythmic transcription [35,36]. The activity of RNA polymerase II (RNA Pol II) might increase the mobility of promoter and enhancer loci in nuclei to facilitate gene expression robustness once transcription is initiated [37]. Moreover, BMAL1 also rhythmically regulates enhancer–enhancer interactions to adjust the timing of gene oscillations in a tissue specific fashion and this process might also involve transcriptional regulators recognizing histone modifications associated with transcriptional activation [38]. Circadian chromatin architecture may also be under the control of additional cycling chromatin remodelers [39]. Moreover, many additional cell-type, tissue-specific and signal-dependent epigenetic regulations exist to tightly control the expression of clock-controlled genes to regulate metabolic outputs and adjust to specific cellular requirements [40] (Figure 1B).

Finally, the heterodimer BMAL1/CLOCK stimulates the expression of the proline and acidic amino acid-rich basic leucine zipper (PAR-bZIP) transcription factor family, including the D site of albumin promoter (*Dbp*), hepatic leukemia factor (*Hlf*), thyrotroph embryonic factor (*Tef*) and antagonist E4 promoter binding protein (*E4bp4,* also repressed by REV-ERBα [41]), which provide clock-related feedback and act through the D-box to control the expression of many circadian clock output genes in peripheral organs [40,42,43]. However, the PAR-bZIP transcription factor family is not necessary for circadian behavior [44].

Studies of plants, fungi, and eubacteria [45,46] suggest that circadian clocks confer an evolutionary advantage by promoting the efficient acquisition and storage of fuel at the appropriate time each day. In mammals, the circadian clock coordinates anabolic and catabolic processes in peripheral tissues with the behavioral sleep–wake and fasting–feeding cycles (described later in “Clock genes, sleep and metabolism”). In mice, approximately 40% of protein-coding genes display rhythmic transcription [27]. A consistently elevated proportion of cycling proteins has also been detected by mass spectrometry in mouse livers [47], whereas ~50% of metabolites display a circadian pattern [48]. Remarkably, in the diurnal baboon, more than 80% of transcripts have rhythmic oscillation (in at least one tissue). A difference between the two species is that the peak phases of expression of core-clock components, except for the master clock, the suprachiasmatic nucleus (SCN), are ~12 h apart, suggesting that nocturnal and diurnal behavior is regulated downstream of the SCN [49].

## 2. Neural Basis of Mammalian Circadian Behavior and Its Interconnection with Sleep and Energy Centers

In mammals, the molecular clock in the hypothalamic SCN is both necessary and sufficient for coordinating daily rhythmic behaviors [50,51,52,53]. Animals with deletions or mutations in genes coding for components of the core-clock display altered lengths of spontaneous activity periods or arrhythmia under conditions of constant darkness (reviewed in [54]). The first demonstrations were performed in the 1990s when the Clock-Δ19 mutation (i.e., 19th exon of the *Clock* gene) was identified in an *N*-ethyl-*N*-nitrosourea mutagenesis screen for mutations affecting circadian behavior in mice [55]. The aforementioned mutation results in a deletion of 51 amino acids in the TAD of the CLOCK protein [56], which can still bind DNA and dimerize with BMAL1; however, the resulting complex is deficient in transactivation [57], thus altering the transcription of clock-controlled genes [58]. SCN graft experiments described the ability to restore normal circadian patterns to arrhythmic animals, with a period determined by the genotype of the grafted tissue (for details, [53]). Mutations in the transcription factors that are able to regulate the expression of neuropeptides critical for SCN intercellular signaling also determine the period of circadian locomotor rhythms in mice [59] or lead to arrhythmia [60].

**Figure 2:**
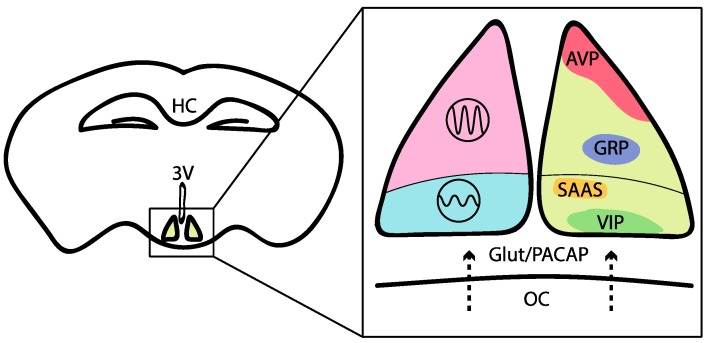
Anatomy of the suprachiasmatic nucleus. A schematic of a coronal slice of the mouse brain showing the location of the suprachiasmatic nucleus (SCN) in the hypothalamus above the optical chiasma (OC). The detailed portion shows the distinct anatomical sections of the SCN. The dorsal and ventral SCN are in pink and blue respectively. Subsets of SCN neurons are shown on the right: GABA^+^ (yellow), VIP^+^ (green), AVP^+^ (red), SAAS^+^ (gold) and GRP^+^ (purple) are expressed in the dorsal SCN. DRD1a^+^ (most SCN cells) and NMS^+^ neurons (~40%) are not shown. HC- Hippocampus, 3V- third ventricle.

The SCN consists of ~20,000 circadian neurons that can be subdivided based on their peptidergic identity (see Figure 2 for details). For instance, neurons expressing vasoactive intestinal peptide (VIP) are in the ventral (core) region of the SCN. These neurons receive direct retinal input through the photosensitive melanopsin neurons, which project via the retinohypothalamic tract [61]. These SCN core neurons exhibit relatively low-amplitude molecular clock rhythms and are readily reset by environmental cues such as light [62,63,64]. SCN neurons expressing the neuropeptide arginine vasopressin (AVP) are mostly located in the dorsal region of the SCN and generate high-amplitude molecular clock rhythms [65,66,67]. Importantly, both peptides are important for proper locomotor rhythms and entrainment to phase shifts, such as jet lag [68,69,70,71,72,73]. For example, *VIP*- or *VIPR*-knockouts result in severe deficits in locomotor activity rhythms, including i) a phase advance in the first day of constant darkness, ii) shorter free-running periods and iii) a lack of response after a light pulse during the subjective night (see for review [72]). Targeting neuromedin S (NMS)-positive cells also impacts circadian behavior. In fact, *Nms* expression is restricted within the SCN; this neuropeptide is produced by ~40% of all SCN neurons, including the majority of AVP and VIP cells [74]. The NMS-neuron-autonomous clock is essential for circadian behavior, and the cells involved act as essential pacemakers in the SCN to couple circadian clock neurons and determine the period of the SCN and behavioral rhythms [74]. Finally, D1 dopamine receptor (DRD1a)-expressing neurons represent most cells in the SCN, including most VIP-positive cells and most AVP-positive cells [75]. Notably, the activation of these neurons is sufficient to entrain circadian behavioral rhythms in mice [75]. Additional nonphotic signals have been less studied, but these signals might, for instance, involve the dopaminergic input (DRD1a-expressing neurons) of the ventral tegmental area to the master circadian clock [76].

A hallmark of most SCN neurons is that they display a 24 h rhythm in neuronal activity that drives daily changes in behavior. It has been observed that the spontaneous firing rate and resting membrane potential of circadian neurons peak during the day/dawn [77] and are circadian clock-dependent [78,79]. These rhythms persist even in dissociated/uncoupled circadian pacemaker neurons in invertebrates (*Bulla*) [80] and in dissociated SCN neurons in vertebrates [81,82]. The cell-autonomous molecular clock generates 24 h of gene expression, and these transcriptional oscillations are linked to neuronal activity rhythms to generate circadian behavioral changes (reviewed in [83]). A remarkable feature is the conserved daily antiphase cycles of sodium and potassium currents driving *Drosophila* and mouse circadian clock neuronal rhythms [84].

Understanding the roles played by the different neuronal cell types in the coupling of the SCN network has been a major challenge. An approach undertaken to address this question has been the overexpression of mutant CLOCK proteins or cell-type-specific ablation of circadian clock genes, for example, in AVP-positive neurons [71] or NMS-positive neurons [74]. Recent observations described that not only neurons but also astrocytes contribute to driving the circadian rhythm, as restoring the astrocyte circadian clock in *Cry1 Cry2* double KO arrhythmic mice is sufficient to restore the circadian rhythm, which is explained by the rescued oscillations of glutamate release by these cells, which are required to reinstate a rhythm in SCN neurons [85,86].

The SCN has many efferent anatomic projections targeting various brain centers, including the hypothalamic centers involved in the regulation of wakefulness, activity, and feeding and projections to the brain stem and the paraventricular nucleus of the hypothalamus to regulate melatonin secretion from the pituitary gland [87]. The hypothalamic neuronal system that links the sleep–wake cycle to the fasting–feeding cycle is, in part, hypocretinergic (the hypocretin peptide is also called orexin) and is responsive to the SCN circadian pacemaker [88,89]. Optogenetic approaches revealed that SCN AVP neurons also project to thirst neurons in the organum vasculosum of the lamina terminalis and mediate the anticipatory surge in water intake prior to sleep [90]. In humans, functional magnetic resonance imaging experiments also demonstrate a relationship between homeostatic sleep pressure and activity in the suprachiasmatic area, suggesting a similar circadian influence on sleep homeostasis [91]. Notably, the SCN has also been reported to play a role in noncircadian social behavior in mice, as VIP and VIPR in this area appear necessary and sufficient for mediating contagious itch [92].

## 3. Clock Genes, Sleep and Metabolism

Clock genes and sleep. In addition to the numerous studies linking clock genes to sleep homeostatic control in animal models (reviewed in [93]), the identification of mutations in the core-clock genes or coding for proteins that regulate the molecular clockwork in humans has led to advances toward understanding the role of clock genes in the regulation of sleep–wake timing, providing a better understanding of their impact on sleep patterns and chronotypes. For example, a specific mutation in *PER2* that affects *the phosphorylation of casein kinases 1* (*CK1ε* and *CK1δ*) of PER2 proteins and consequently modulates the core circadian protein turnover by triggering proteasomal degradation of PER2 (see Figure 1A), or mutations of *CK1δ* itself, may result in familial advanced sleep phase disorder (FASPD) [94,95,96]. Patients with FASPD display abnormal sleep patterns characterized by excessive tiredness in the late afternoon, very early waking times and eventually insomnia. Similarly, a mutation altering the conformation of CRY2, increasing its accessibility to and affinity for FBXL3 (an E3 ubiquitin ligase) (see Figure 1A), thus promoting its degradation, has also been described to result in FASPD [97]. An advanced sleep phase has also been associated with polymorphisms of the *PER1* gene [98].

In contrast, a case of familial delayed sleep phase disorder (DSPD), which is characterized by a persistent and intractable delay of sleep onset and offset times relative to the societal norm, has been linked to a dominant coding variation in *CRY1* [99]. In this study, the allele was shown to encode a CRY1 protein with an internal deletion, affecting its function as a transcriptional inhibitor and lengthening the circadian period [99]. Additional findings indicate that several coding variations of the core-clock gene *PER3* may be associated with late chronotype and DSPD patterns [100,101,102,103,104]; however, these studies relied on limited samples and/or may be population-specific. Finally, another example is the association between a specific mutation in the transcriptional repressor DEC2 [105] and a short sleep phenotype [106]. However, in these works, very little or no information described and characterized the metabolic profiles of the studied patients. In one study, some patients with sleep disorders were excluded from the analysis after data collection because of cosegregation of aberrant sleep in carriers with morbid obesity [99].

**Figure 3:**
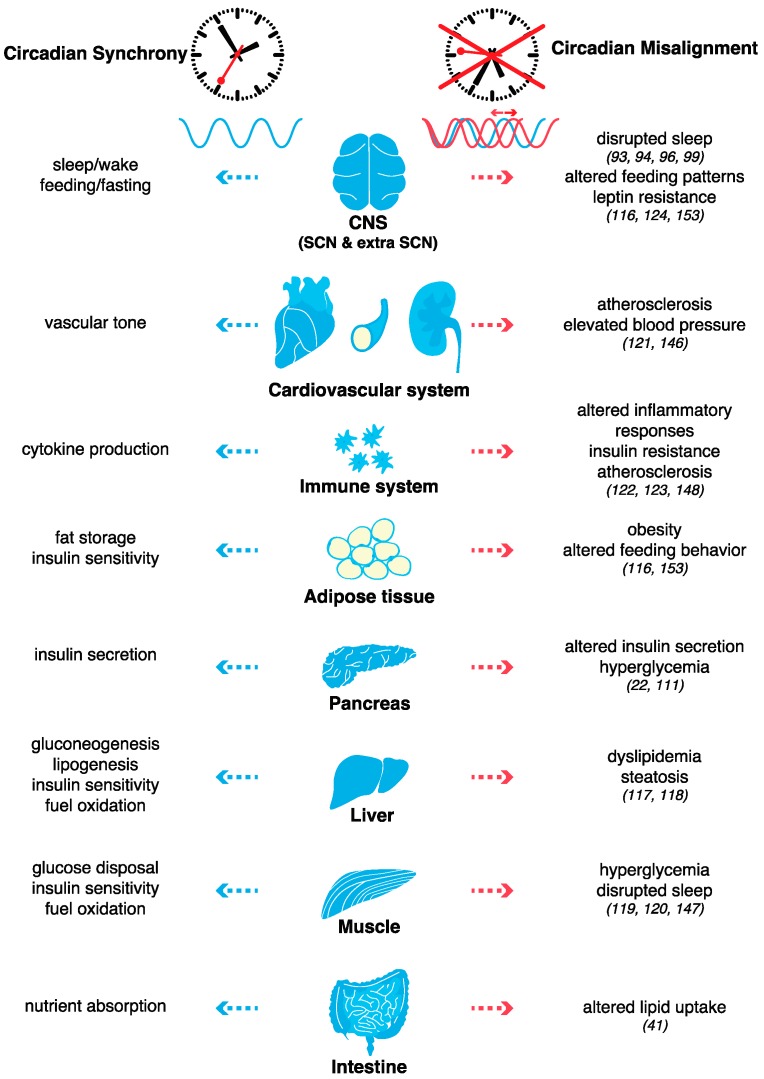
From circadian synchrony in physiological processes to circadian disruption in metabolic diseases. In mammals, circadian clocks are found in all major organ and tissues. The mammalian circadian pacemaker is located in the suprachiasmatic nucleus (SCN) of the hypothalamus. The SCN coordinates all of the cell autonomous oscillations in the peripheral tissues to maintain robustly rhythmic behavioral and physiological phenotypes. The coordination between behavioral (i.e., sleep–wake, feeding–fasting) and metabolic responses with the light/dark cycle involves the autonomic innervation and/or endocrine signals. During circadian misalignment induced by chronic jet lag, irregular eating times, high-fat feeding, abnormal sleep patterns, the peripheral tissues and the brain fail to receive the appropriate signals at the optimal time of the day, thereby potentiating the development of metabolic diseases. The main references (studies in mice and/or humans) are indicated.

From circadian clocks to metabolism: lessons from genetic models and human polymorphisms. A transformation in the understanding of circadian clock function occurred with the following discoveries that were made two decades ago: the heterodimer CLOCK/BMAL1 oscillates within not only the master pacemaker neurons of the SCN but also within peripheral cells and circadian oscillations occur even in isolated cells ex vivo following synchronization via serum, temperature, or cyclase agonists [107]. In fact, peripheral organs (especially the liver) have even more cycling genes than the hypothalamus [27]. In addition, with the first observations that peripheral cells could display self-sustained cellular circadian oscillators [107], the ideas that external cues such as feeding cycles or jet lag could disrupt these oscillators [108,109] and that either environmental or genetic perturbations of the molecular clocks may lead to metabolic disorders were originated [110]. The discovery that the *Clock*-mutant mouse is susceptible to obesity transformed our understanding of the molecular link between circadian clocks and metabolism [110]. In addition to disruptions in sleep and circadian behavior, these mice also develop hyperphagia early in life, with the subsequent emergence of hyperlipidemia, hyperleptinemia, and hypoinsulinemic hyperglycemia, a hallmark of endocrine pancreas dysfunction [110,111]. Similarly, *Bmal1* KO, *Per2* KO, and *Cry1 Cry2* double KO result in susceptibility to obesity and metabolic disorders [112,113,114,115]. Abrogation of the molecular clock restricted to adipose-tissue [116,117], pancreas [22,111], liver [113,118,119], skeletal muscle [120,121], smooth-muscle [122], myeloid cells [123,124] or even arcuate nucleus AgRP neurons [125] further confirmed this link (Figure 3). 

In agreement with these findings, in humans, polymorphisms in several clock genes have been linked to obesity or to other features of metabolic syndrome (reviewed in [126]) and cardiovascular consequences [127]. Observational studies in small sample populations have shown that polymorphisms in the *CLOCK* gene are associated with predisposition to obesity [128,129], and that two single nucleotide polymorphisms in *BMAL1* are associated with Type 2 Diabetes (T2D) and hypertension [130]. Moreover, several studies have described an association between polymorphisms in *CRY2* and elevated fasting glucose [131,132]. An association between polymorphisms in REV-ERBα and obesity was found only in men [133]. Interestingly, it has also been reported that environmental factors such as nutrient intake and sleep duration may modify the cardiometabolic abnormalities conferred by common circadian-related genetic variants [134]. Finally, in addition to the core-clock gene machinery, several genome-wide association studies have revealed that melatonin, a hormone secreted by the pituitary gland that is implicated in circadian rhythms, may be important in the regulation of glycemia. In particular, these studies described the impact of the *MTNR1B* (melatonin receptor 1B) polymorphism on fasting glucose or insulin-secretion-related traits and on T2D risk [135,136,137,138], which might be explained by the impact of melatonin signaling on insulin secretion [139]. Notably, the melatonin profile relative to the feeding-fasting cycle is reversed when individuals are subjected to forced desynchrony [140].

From altered sleep and/or circadian misalignment to metabolism. Another set of important observations has revealed that, in humans, environmentally induced alterations in the sleep–wake cycle exert consequences on metabolic fitness. In particular, sleep deprivation and poor sleep quality are significantly associated with an elevated risk of obesity, diabetes, and other metabolic diseases (reviewed in [88]). Recent genome-wide association studies have also reported several loci for insomnia symptoms and highlighted shared genetics with metabolic traits [141,142,143]. Interestingly, pathway and ontology analyses have revealed a role of ubiquitin-mediated proteolysis, in accordance with previous evidence from experimental genetic models, describing their involvement in sleep/circadian rhythms [142]. Another striking example is the impact of sleep deprivation on the pathogenesis of cardiovascular diseases. Atherosclerosis has been independently associated with short sleep duration in humans [144], which might be explained by the low hypocretin levels associated with sleep deprivation, the causal effect of the increased production of monocytes by the bone marrow and the stimulation of the maturation of monocytes into macrophages, as shown in mice [145]. 

To gain a better understanding of the impact of external perturbations on internal circadian rhythms and metabolism, studies with controlled laboratory conditions designed to simulate shift work were performed. Remarkably, the results demonstrated that the misalignment of the endogenous circadian system with external time appears to play an even larger role than short sleep duration in the pathogenesis of metabolic and cardiovascular diseases [146]. Participants who were subjected to such a forced misalignment were described as having decreased insulin sensitivity and increased inflammation compared with the insulin sensitivity and inflammation of the study participants who maintained regular nocturnal bedtimes with identical total sleep durations [146]. Even a short-term circadian misalignment for a 1-week period is sufficient to increase 24-h blood pressure and inflammatory markers in healthy adults [147]. More recently, two studies described that almost half of the human plasma proteome shows variations specific to the time of the day and that a short period of simulated shift work significantly affects a fraction of these diurnal changes [148,149], as well as changes in glucose, free fatty acid and triglyceride patterns [148]. However, although some genome-wide association studies in large cohorts have found positive associations between chronotype (i.e., innate preference for mornings or evenings) and body mass index and/or other metabolic parameters, these studies failed to find a clear causal relationship [150,151,152,153]. Collectively, these observations suggest that circadian misalignment, rather than the chronotype itself, is more strongly associated with disease outcomes (Figure 3).

Because of the pervasive impact of circadian disruptions and the difficulty of evaluating their full effect in humans, animal models have also provided useful information regarding the impact of environmentally induced circadian disruptions (e.g., chronic jet lag). Such experiments confirmed that, similar to genetic circadian clock models, environmentally induced misalignment also leads to obesity and that leptin resistance might be a common hallmark of circadian misalignment [117] (Figure 3). Another similarity between genetic and environmentally induced circadian clock disruptions is the change in a large subset of the gut microbiome [154], which in turn, may modulate the intestinal epithelium and exert subsequent effects on the host metabolism [155] as well as on transcriptional and epigenetic programs [156] (reviewed in [157]).

The circadian clocks sense metabolic cues. A surprising finding was that circadian disruption could emerge not only from altered sleep patterns, jet lag, or clock gene polymorphisms but could also be induced by both the timing of food intake and macronutrients, and peripheral clocks, especially, are highly sensitive to these cues [158,159,160]. Although the phase of the SCN appears to be resilient to external perturbations such as temperature rhythms or glucocorticoids, potent synchronizers of peripheral tissues [161,162], several lines of evidence now suggest that the circadian cycle modulates nutrient homeostasis through bidirectional communication between peripheral tissues and the brain and that metabolic cues modulated by the diet might also act on the SCN. For example, an ad libitum obesogenic diet exerts especially deleterious effects on circadian systems since animals fed such a diet shift their pattern of food intake such that they consume more calories during the ‘incorrect’ circadian time (i.e., during the light period) [158]. Remarkably, feeding a high-fat diet (HFD) during only the light period exacerbates diet-induced obesity compared to feeding the equivalent amount (same energy intake) of fat during the dark period [160]. Conversely, restricting access to high-fat food to the ‘correct’ circadian time (the dark period) ameliorates diet-induced metabolic syndrome [163,164]. Importantly, time-restricted feeding may also lead to metabolic improvements in obese patients with prediabetes [165]. Moreover, restricting high fat intake to the night has also recently been reported to improve metabolic health in young mice with a compromised circadian clock, suggesting that improved lifestyle management by promoting time-restricted eating in populations with dampened circadian rhythms, such as shift workers, might improve metabolic fitness [166]. 

On the other hand, besides high-fat feeding experiments, caloric restriction studies also indicate that the feeding cues might target the SCN. For example, it has been reported that caloric restriction might reset the SCN clock even without synchronization to daily meal timing [167]. In fact, several studies demonstrated that hypocaloric feeding affects SCN clock gene expression in both diurnal and nocturnal mammals [168,169]. Moreover, a recent study described that caloric restriction dramatically alters locomotor activity in mice. In particular, mice under caloric restriction choose their own feeding times and consolidated their food intake to the nighttime while enhancing locomotor activity during the resting phase [170], furthering this hypothesis. Collectively, these studies with either dietary fatty acid overflow or limited calorie intake suggest that peripheral metabolism modulated by the nutritional status might feedback to the master clock (see Table 1, further detailed later). 

Mechanistically, both a HFD and restricting caloric intake (without inducing malnutrition) impact a great amount of metabolites and diverse signaling pathways. In the liver, a HFD leads to three major changes in the circadian metabolome: loss of metabolite cycling, de novo metabolite cycling, and a phase shift of a subset of metabolite oscillations [171]. Such changes following HFD feeding are associated with a decreased recruitment of BMAL1 and CLOCK to the promoters of usual clock-controlled genes, thus showing dampened rhythmic patterns of expression with a HFD, while de novo rhythmic patterns of expression have been reported to be mediated by a mechanism that is dependent on rhythmic activation of PPARγ [171]. The acquisition of rhythmicity for the noncore clock components PPARα and SREBP1 also remodels the metabolic gene transcription response to HFD feeding [172].

Another pathway linking HFD feeding to circadian disruption is the IKK/ NF-κB inflammatory signaling pathway. Many of the complications of HFD feeding result in part from the state of chronic low-grade inflammation induced by the activation of IKK signaling and NF-κB-mediated transcription within both the hypothalamus and peripheral tissues such as the liver and fat [173,174,175]. In the liver, the p65 subunit of NF-κB regulates the expression of the negative limb of the clock feedback loop by directly antagonizing CLOCK/BMAL1 on the *Per, Cry* or *Rev-erb* promoters [18]. Interestingly, HFD feeding also redistributes p65 genome-wide, which might act as a pioneering transcription factor by promoting chromatin accessibility to relocalize CLOCK/BMAL1-binding sites in its close proximity, together with acetylated H3K27, a marker of transcriptionally active chromatin, and RNA Pol II following HFD feeding [18]. Whether p65 is first to engage target sites in chromatin to causally determine DNA accessibility and initiate cooperative interactions with other reprogramming factors [176,177] might be further validated by ATAC-seq approaches in HFD-fed mice. HFD-induced hepatic activation of both NF-κB and PPARγ might be mediated by gut microbiota components (e.g., lipopolysaccharides (LPS)) or their fermentation products (e.g., short-chain fatty acids (SCFAs)), respectively, and are associated with genes involved in nutrient-responsive anabolic pathways, especially lipid metabolism and lipid accumulation [18,178,179,180]. Such pathways can be induced quickly after only a few days on a HFD and are also important for the normal physiological function of mice fed a regular chow diet, suggesting that these pathways play a key role in circadian homeostasis and metabolic fitness and reinforcing the necessity for a better understanding of such regulatory pathways to use them as optimal targets to prevent hepatic metabolic alterations. However, a caveat has been that the “reprogramming” effects of a HFD have been mainly studied in mouse livers, while the most significant effects of a HFD on the amplitude of core-clock and overall clock-controlled gene expression have been found in mouse adipose tissue [18,158]. As expected, recent data showed that adipose tissue from HFD-fed mice also display a decreased recruitment of BMAL1 at the promoters of some clock-controlled genes [181]. However, adipocyte-specific deletion of *Bmal1* modifies not only adipose physiology but also the function of hypothalamic neurons involved in appetite regulation, resulting in a shift of feeding patterns [116]. Thus, it would be interesting to further explore the reorganization of circadian rhythm by nutrients in adipose tissue, by identifying the specific signals and pathways involved in adipose clock reprogramming following a HFD challenge and by examining the associated changes in the binding of specific transcription factors, in order to determine the subsequent impairment in tissue function. Moreover, it would be important to determine to what extent these findings in mice can be extrapolated to humans. 

A recent study further analyzed 24 h metabolite profiles of 8 tissues from HFD-fed mice and revealed that HFD feeding actually rewires the circadian metabolism in several tissues, although temporal variations in metabolites in peripheral tissues were more impacted than the SCN [182]. HFD feeding led to alterations in ~30% of SCN metabolites, while ~40–60% of metabolites were dysregulated in peripheral tissues such as adipose, muscle and liver tissues, and the changes concerned mostly specific lipid classes (including long-chain fatty acids (LCFAs), polyunsaturated fatty acids (PUFAs), and diacylglycerol (DAG)) and metabolites involved in amino acid metabolism [182]. Importantly, temporal metabolite communication between these tissues is compromised by HFD feeding, and the subsequent desynchrony might also be involved in the pathogenesis of T2D and cardiovascular diseases [182]. Collectively, these observations suggest that in the case of the internal misalignment induced by HFD feeding, in a similar way to the one induced by chronic jet-lag, the organs fail to receive the appropriate signals at the optimal time of the day, thereby potentiating the development of disease states.

Interestingly, the impact of HFD feeding is tissue-specific, and a surprising observation from this study is the induction of ~100 de novo oscillating lipids to the SCN, while by comparison, another brain region (the medial prefrontal cortex) lost oscillating lipids [182]. Whether such lipid changes impact CLOCK and BMAL1 binding at the chromatin level either in pacemaker neurons or in astrocytes, as well as the induction of surrogate transcriptional regulatory pathways in these cells, needs to be further explored. Another pathway, the IKK/NF-κB pathway linking overnutrition to circadian disruption, might play an important regulatory role in the central clock in the SCN. Indeed, the abrogation of IKK/NF-κB signaling during adulthood alters *Per2* expression in the hypothalamus, shortens the circadian period and increases daytime locomotor activity, suggesting that it could be an attractive target in the context of metabolic alterations associated with environmentally induced circadian disruptions, such as jet lag and shift work [18]. In the SCN, the interplay between NF-κB and the circadian clock might also be relevant for fever or sickness-induced sleep considering the strong link between these events [183,184].

Emerging evidence indicates that several peripheral metabolic signals, such as NAD^+^ metabolites, influence brain, behavior, and sleep. In mice, CLOCK/BMAL1 regulates NAD^+^ levels through the control of nicotinamide mononucleotide phosphoribosyl transferase (NAMPT), a rate-limiting NAD^+^ biosynthetic enzyme within peripheral tissues [32,33]. The direct activation of *Nampt* by CLOCK and BMAL1 leads to elevated NAD^+^ levels, increased activity of the NAD^+^-dependent deacetylase SIRT1 and, subsequently, reduced CLOCK/BMAL1 activity, as SIRT1 is an inhibitor of the positive limb of the clock feedback loop. SIRT1 is expressed in several hypothalamic nuclei and is mainly involved in normal energy expenditure adaptations to prevent diet-induced obesity [202]. SIRT1 is also a key nutrient sensor [203], and in the SCN, SIRT1 plays a critical role in the molecular integration of metabolism and circadian rhythms, which can directly activate *Bmal1* expression [204]. NAMPT and its product nicotinamide mononucleotide (NMN) are produced in peripheral tissues, circulate in the blood and are delivered to the brain [194,195]. In addition to SIRT1, poly (ADP-ribose) polymerase 1 (PARP-1) is also an enzyme whose activity depends on NAD^+^ levels, and PARP-1 is able to regulate the circadian clock. PARP-1 oscillates in a daily manner, and poly(ADP-ribosyl)ates bind CLOCK at the beginning of the light phase, thereby reducing the DNA binding activity of CLOCK/BMAL1 [205]. Additional molecular regulatory pathways link nutrient sensing and metabolism with the core circadian clock. Another mechanism involves circadian regulation via AMP-activated protein kinase (AMPK) signaling, a pathway activated by decreased ATP production [and increases in adenosine monophosphate (AMP) production]. The impact of AMPK on the circadian clock is in part mediated by the degradation of the core-clock repressor, CRY1 [206] (Figure 1A).

Among the peripheral metabolic signals regulated by nutritional status and able to modulate the central clock in the SCN is fibroblast growth factor 21 (FGF21) [189] (Table 1). FGF21 is a hepatokine that is upregulated by a ketogenic diet (or fasting). FGF21 acts on β-Klotho, which is known to form a complex with FGF21 receptors expressed in the SCN to mediate its effects on the modulation of wheel running behavior, reduce insulin levels and increase corticosterone levels [189]. Interestingly, liver-derived ketone bodies can also target the brain to induce food anticipation behavior [207].

Other peripheral organs release metabolites and endocrine factors (Table 1). Adipose tissue secretes a great source of endocrine peptides called adipokines. Among the adipokines, leptin, whose systemic levels correlate with adipose tissue mass but also reflect immediate changes in nutritional status as these levels decrease quickly upon fasting, acts as a true hormone to control bodily energy balance [208]. Leptin receptors are also found in the SCN [186], which might account for some of its effects on the regulation of sleep–wake cycles [187,209]. Moreover, leptin activates the production of anorexigenic neuropeptides such as α-melanocyte-stimulating hormone/cocaine- and amphetamine-regulated transcript (αMSH/CART), which in turn blocks the production of the orexigenic peptide hypocretin [89]. Hypocretin signaling, which is decreased during sleep deprivation [145], also has the capacity to primarily promote energy expenditure via leptin sensitization [210]. Notably, the leptin-responsive JAK-STAT signaling pathway also displays a diurnal rhythmicity in order to coordinate the leptin response and glucose metabolism with arousal [125]. Ghrelin is a stomach-derived hormone able to stimulate locomotor activity in anticipation of meals in mice [190]. Ghrelin has been reported to reset the master clock in vitro [193] and to increase slow-wave sleep [192], which might be explained by its inhibitory effects on the activity of hypocretin neurons [191]. Thus, ghrelin might play an important role in the control of circadian behavior and sleep architecture in addition to its orexigenic effect.

Several metabolites produced by *skeletal* muscle and myokines have been suspected to regulate diurnal behavior (Table 1). Interestingly, the *Bmal1* gene in skeletal muscle regulates responses to sleep deprivation [120], which might be explained by the altered 24 h production of myokines or muscle metabolites. For example, kynurenine, whose plasma levels are reduced with exercise, is able to cross the blood-brain barrier, and decreased kynurenine levels prevent the stress-induced neurobiological mechanisms of depression [188]. The muscle-derived peptide irisin can also be released into circulation [211], especially under conditions of saturated fatty acid overload [212]. Irisin, in turn, exerts central effects [196,197]. Exercise also leads to the release of amino acids and lactate into circulation. Lactate, which is also increased in the fasting state, can be used as a main fuel [213] and reach the brain [214]. Lactate has been reported to modulate the DNA binding of CLOCK/BMAL1 and NPAS2/BMAL1 heterodimers [199]. Astrocytic lactate release has been described to regulate the activity of orexin neurons and the regulation of sleep-wake cycles [198].

Many other metabolites or hormones, including insulin [200], can act as a cue for the circadian clock. Insulin stimulates pro-opiomelanocortin (POMC) neurons [201] and might also play a role in the regulation of the sleep–wake cycle [185]. Glucose (as well as glutamine) is a major sensor because it can be converted to uridine diphosphate *N*-acetylglucosamine (UDP-GlcNAc) through the hexosamine biosynthetic pathway, which is catalyzed by *O*-GlcNAc transferase in β-d-*N*-acetylglucosamine *O*-linked to polypeptides (a process called *O*-GlcNAcylation). In turn, *O*-GlcNAcylation reversibly modifies CLOCK and PER2 to regulate their transcriptional activities and circadian period length [215]. This phenomenon is conserved across species as it occurs both in mouse brains and *Drosophila* neurons. Moreover, high glucose levels also increase the *O*-GlcNAcylation of a region in PER2 that is known to regulate the human sleep phase by competing with the phosphorylation of this region [215]. BMAL1 can also receive such posttranscriptional modifications, at least in the liver [216]. Interestingly, a recent study completed these observations by showing that REV-ERBα also rhythmically binds to *O*-GlcNAc transferase to regulate ten-of-eleven translocation (TET) enzymes and DNA hydroxymethylated cytosine (5hmC) levels in the vicinity of REV-ERBα genomic binding sites, reciprocally [217].

In addition to these metabolic sensors, oxygen, via hypoxia-inducible factor 1α (HIF1α) activation, is also a resetting cue for circadian clocks [23,218,219,220]. Remarkably, nutrient ingestion is also tightly linked to oxygen consumption, and restricted feeding restores tissue oxygenation rhythmicity in circadian clock-deficient mice [219]. A well-designed study found that tissue oxygenation exhibits daily rhythms and that peripheral oscillators are sensitive to variations in oxygen levels within the physiological range [218]. This study further described that oxygen levels influence the expression of core-clock genes in an HIF1α-dependent manner in the brain [218], which is consistent with the impact of HIF1α-stabilizing treatments on PER2::LUC period-lengthening and amplitude-reducing oscillations in the SCN [23]. Finally, a low oxygen pulse has also been reported to accelerate the adaptation to jet lag in mice [218]. The interplay between oxygen and circadian clocks has been explained by the impact of HIF1α on the circadian clock [218]. Interestingly, there is a great deal of overlap between BMAL1 and HIF1α cistromes [23]. Indeed, HIF1α belongs to the bHLH-PAS transcription factor superfamily and can be recruited to the *PER2* promoter to activate its transcription, resulting in the disruption of the circadian system. Because IKK/NF-κB is an important physiological contributor to the hypoxic response, linking it to innate immunity and inflammation by playing a key role in HIF1α accumulation under hypoxic conditions [221], it would be relevant to further examine the link between these pathways in the regulation of circadian behavior.

## 4. The Emerging Field of Chrono-Pharmacology

In addition to highlighting the importance of proper coordination of circadian behavior, sleep homeostasis, and cell metabolism to preserve metabolic fitness (Figure 3), recent observations also highlight the advantage of a better understanding of human circadian clock coordination between different tissues for emerging fields such as chrono-medicine or chrono-pharmacology. Because a large proportion of coding genes are expressed in a circadian fashion and because cardiometabolic disease might arise, at least in part, from circadian disruptions, chrono-medicine or chrono-pharmacology might be appropriate to prevent (and treat) the pathogenesis of cardiometabolic diseases. Mathematical models have played an important role in the research of circadian rhythms of gene expression, proteins and metabolites [222,223]. Algorithms that robustly infer circadian time from gene expression have been generated to improve the diagnosis of circadian disorders and optimize the delivery time of therapeutic treatments [224]. By using an algorithm to detect cycling gene expression in biopsies collected from >600 human donors, a study demonstrated that nearly half of protein-coding genes were cycling in at least 1 of the 13 tissues analyzed [225]. Focusing on the cardiovascular system, these analyses allowed the identification of 1000 cycling genes encoding proteins that either transport or metabolize drugs or are themselves drug targets and to connect more than 100 drugs to 136 genes oscillating robustly in at least one atrial chamber, aorta, coronary artery, or tibial artery, paving the way for timed administration of drugs in pursuit of circadian medicine for cardiovascular disease [225]. In parallel, some experiments already obtained impressive results in mice. For example, studies in high-fat fed mice have shown that the development of atherosclerosis is controlled by the circadian system and that circadian myeloid cell recruitment to the arterial wall sustains atherogenic growth [123]. Thus, atherosclerosis might be more efficiently managed with the timing of drug delivery [123]. Whether these results translate well to humans still needs to be confirmed, but these findings are opening a new avenue to test the preservation of metabolic homeostasis by chrono-pharmacology in subjects with disrupted circadian rhythms.

Taken together, these studies demonstrate that in addition to the erratic lifestyle associated with modern society, such as jet lag or shift work, altered feeding patterns and nutrient-induced metabolic cues can also disrupt circadian rhythms. Evidence demonstrates that disruptions in the temporal coordination of metabolism and physiology induced by high-fat food consumption promote the pathogenesis of cardiometabolic disease (Figure 3). Lifestyle improvements to correct feeding patterns such as time-restricted feeding [163,164] and pharmacological treatments to enhance circadian clock function [226] or chrono-pharmacology [123] have been shown to improve metabolic fitness.

## 5. Conclusions

The rising incidence of obesity and cardiometabolic disease in recent decades poses a serious public health concern in Europe, the US, and the developing world, and disturbances in circadian rhythms contribute to this increasing incidence. Metabolic fitness in animals and humans involves interactions between environmental and genetic factors and, among these factors, the cellular circadian clock is unique in generating daily rhythms of sleep, feeding, and metabolism in synchrony with the environmental light cycle. Genetic inactivation of the core clock results in obesity and cardiometabolic disorders, suggesting that misalignment between internal circadian oscillators and/or between endogenous and environmental rhythms may lead to reduced metabolic fitness, which has now been confirmed by translational studies. Collectively, a better knowledge of the mechanisms by which circadian clock function can be compromised will lead to novel therapeutic interventions for T2D, obesity, and other metabolic disorders arising from circadian disruption.

## Figures and Tables

**Table 1:** Examples of endocrine signals relaying nutritional status from the periphery to the brain, with potential or established effects on the regulation of circadian rhythms and/or sleep.

Protein or Metabolite	Main Peripheral Sources	Main References
Leptin	Adipose tissue (adipocytes)	[117,125,185,186,187]
Kynurenine	Liver (hepatocytes)	[120,188]
FGF21	Liver (hepatocytes)	[189]
Ghrelin	Stomach (parietal cells)	[190,191,192,193]
NAMPT	Peripheral tissues, adipose tissue	[194,195]
Irisin	Skeletal muscle	[120,196,197]
Lactate	Skeletal muscle	[198,199]
Insulin	Pancreas (β cells)	[185,200,201]
